# Acute Kidney Injury Due to Ureteral Damage by Needle-Shaped Crystals Associated With Boron Neutron Capture Therapy

**DOI:** 10.7759/cureus.76094

**Published:** 2024-12-20

**Authors:** Akira Mima, Tatsumasa Matsuki, Takahiro Nakamoto, Yuta Saito, Takaaki Morikawa, Sakura Kure, Shinji Lee

**Affiliations:** 1 Nephrology, Osaka Medical and Pharmaceutical University, Takatsuki, JPN

**Keywords:** acute kidney injury, boron neutron capture therapy, needle-shaped crystal, reactive oxygen species, ureteral damage

## Abstract

A 77-year-old man was referred to our department because of macrohematuria, oliguria, and a serum creatinine level of 2.47 mg/dL during boron neutron capture therapy (BNCT) for oropharyngeal cancer. At baseline, his creatinine level had been 0.98 mg/dL. The vital signs and physical examination were normal. A urinalysis showed protein and numerous red cells per high-power field, and needle-shaped crystals were observed. A plane computed tomography showed hematoma within the extensive ureter. However, hydronephrosis was not recognized. Immediate discontinuation of BNCT and supplemental fluids reduced gross hematuria, increased urine, and creatinine decreased to 0.97 mg/dL. BNCT is an innovative radiation therapy that targets tumor cells by inducing a nuclear reaction between 10B and neutrons within the tumor. However, there have been no reported cases of treatment-related boron crystals causing ureteral injury that leads to acute kidney injury, and oncologists should be aware of this potential risk.

## Introduction

Boron neutron capture therapy (BNCT) is an innovative cancer treatment in which boron-10 (10B) is selectively delivered to cancer cells. These cells are then exposed to neutrons of the appropriate energy, triggering a nuclear reaction that releases lithium-7 (7Li) nuclei, which are high linear energy transfer (LET) particles [1].

Although BNCT is a safe treatment that allows high-dose irradiation of tumors with low doses to normal tissues, the target patients are often those with lower reserve capacity than usual [2]. BNCT is not indicated because renal failure cases were not included at the time of the trial. BNCT may induce renal damage because of the high dose of boron used during BNCT treatment, 500 mg/kg [3]. In fact, there appear to be reports of gross hematuria and acute kidney injury (AKI), which is a relatively new concept in renal impairment, but these have not been published in the form of case reports. This paper is the first to identify urinary crystals as a cause of gross hematuria associated with BNCT therapy and the associated acute kidney injury.

## Case presentation

A 77-year-old Japanese man was referred to our department with proteinuria, gross hematuria, and oliguria. He had a medical history of oropharyngeal cancer, but his family history was unremarkable for any related conditions. On physical examination, there was no leg edema, the abdomen was soft and tender, and the spleen and liver were normal. The patient showed oliguria and a serum creatinine level of 2.47 mg/dL. Table 1 indicates the clinical data on medical examination.

**Table 1:** Laboratory findings CRP: C-reactive protein, AST: aspartate aminotransferase, ALT: alanine aminotransferase, LDH: lactate dehydrogenase, ALP: alkaline phosphatase, BUN: blood urea nitrogen, Na: serum sodium, K: serum potassium

Laboratory data	Patient’s values	Reference values
White blood cells (10^3^/micro-liter)	5.96	3.1-8.4 (10^3^/micro-liter)
Hemoglobin (g/dL)	10.4	13.0-16.6 (g/dL)
Platelet (10^3^/micro-liter)	160	15-35 (10^3^/micro-liter)
CRP (mg/dL)	2.66	<0.14 (mg/dL)
Total bilirubin (mg/dL)	0.7	0.4-1.5 (mg/dL)
AST (IU/L)	16	<30 (IU/L)
ALT (IU/L)	7	<30 (IU/L)
LDH (IU/L)	148	120-220 (IU/L)
ALP (IU/L)	51	80-260 (IU/L)
Total protein (g/dL)	5.2	6.5-8.0 (g/dL)
Albumin (g/dL)	3.0	4.0-5.0 (g/dL)
BUN (mg/dL)	16	8-20 (mg/dL)
Creatinine (mg/dL)	2.47	0.65-1.09 (mg/dL)
Na (mEq/L)	146	137-147 (mEq/L)
K (mEq/L)	4.5	3.5-5.0 (mEq/L)
Urinalysis		
pH	8.0	4.8-7.5
Blood	3+	-
Protein	2+	-

**Figure 1:**
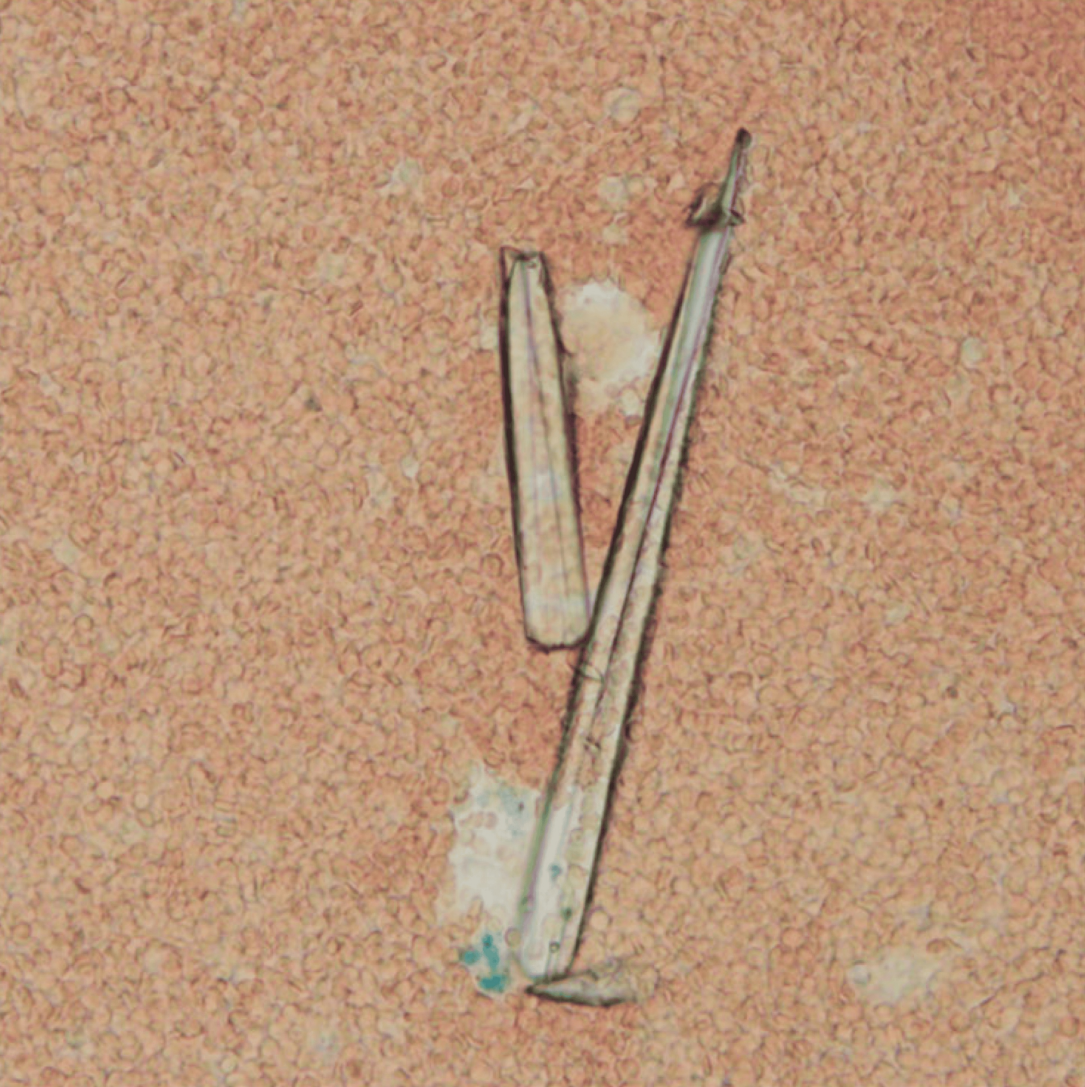
Microscopic urinalysis Needle-shaped crystals were observed in urine under a microscope (original magnification ×400).

Upon examination, numerous red cells per high-power field and needle-shaped crystals were observed (Figure 1).

**Figure 2:**
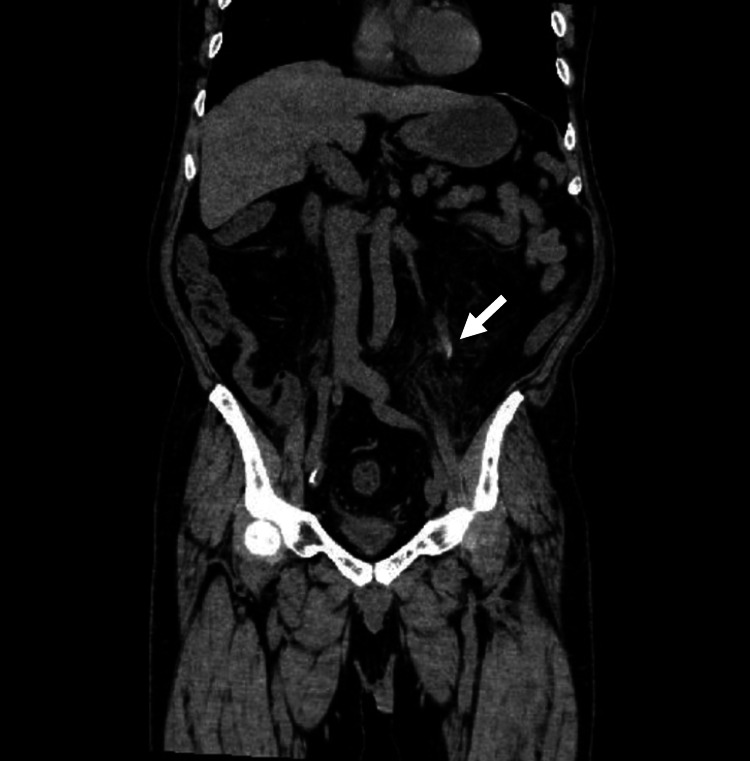
A coronal plane of computed tomography Computed tomography showed hematoma within the extensive ureter (white arrow).

A coronal plane of computed tomography showed hematoma within the extensive ureter. However, hydronephrosis was not recognized (Figure 2).

The patient was diagnosed with acute kidney injury due to ureteral injury and hematoma caused by needle crystals due to boron. Serum creatinine was restored to 0.97 mg/dL by administering a saline infusion. Renal function remained stable and unchanged. In addition, hematuria disappeared and was never seen again.

## Discussion

BNCT has attracted worldwide attention recently, and new researchers have been involved in the development of boron pharmaceuticals. It has shown efficacy primarily in the treatment of head and neck cancer, and its potential is promising [4]. On the other hand, few mechanisms of side effects, especially acute kidney injury, have been reported. Thus, we report here a case of boron-induced urinary tract crystal formation resulting in ureteral injury, gross hematuria and hematoma, and consequently acute kidney injury.

Interestingly, boron, in moderate amounts, has been shown to have the potential to prevent urinary tract stones by exhibiting antioxidant properties [5]. We have shown that reactive oxygen species (ROS) levels are increased in the retina and kidney under diabetic conditions [6-8]. Furthermore, lipid peroxides, isoprostanes, and hydroxydeoxyguanosine are increased in diabetic rodents. Therefore, increases in ROS production in diabetes could be due to abnormal free fatty acid and glucose metabolism. This phenomenon may account for the elevated oxidative stress in non-diabetic insulin-resistant patients. In fact, it has been reported that urinary tract stones are more common in diabetic nephropathy, where such oxidative stress is high [9,10].

Previous reports indicated that oxidative stress and antiproliferative effects occurred in melanoma cell lines in BNCT; melanoma cells were treated with different concentrations of boronophenylalanine and irradiated with thermal neutron flux. BNCT-induced inhibition of free radical production and proliferative capacity of melanoma cells [11]. In other words, the organism is exposed to an excessive state of oxidative stress during BNCT.

AKI leads to chronic kidney disease (CKD) and increases all-cause mortality. In this regard, AKI by BNCT is a serious concern. In order to more accurately diagnose AKI, a new concept has been proposed by KDIGO (kidney disease improving global outcomes), which is based on risk, damage, failure, loss of function, end-stage renal disease (RIFLE) classification, and the acute kidney injury network (AKIN) criteria, as modified [12,13]. Using the KDIGO concept, early diagnosis and appropriate treatment of AKI caused by urinary tract crystals due to BNCT could prevent the number of CKD patients from increasing.

We should recognize that BNCT is not always a safe treatment in terms of renal function. On the other hand, it is currently difficult to predict renal dysfunction caused by BNCT. Therefore, prophylactic measures such as adequate fluid replacement are important. X-ray analysis is required to accurately analyze the boron crystals in urine, but this was not performed at this time. This point is considered a limitation of this case report.

## Conclusions

We experienced a case of AKI due to the formation of needle-like urinary crystals caused by BNCT. Although renal function was restored with supplemental fluids, oncologists need to be aware that BNCT is not always safe.
